# 

**Published:** 1887-11

**Authors:** 


					﻿Volume LV.]
NOVEMBER, 1887.
[Number 5.
's5v'-
MEDICAL}
■ Journal
\^Z	an d
EXAMINER
EDITOR:
S. J. JONES, M.D., LL.D
I'UII I II M • « 11 • • u I I» IH UH f' > »,f	Hf
Clark & longley (q, Chicago,
i '	Pwnr	I.
nlillHHiiiiuifiii mu.,,,.... A. wDLl^OIu I ihh i n i	B
				

